# What Drives Bird Vision? Bill Control and Predator Detection Overshadow Flight

**DOI:** 10.3389/fnins.2017.00619

**Published:** 2017-11-07

**Authors:** Graham R. Martin

**Affiliations:** School of Biosciences, University of Birmingham, Birmingham, United Kingdom

**Keywords:** visual field, retina, fovea, area, foraging, optic flow, binocularity

## Abstract

Although flight is regarded as a key behavior of birds this review argues that the perceptual demands for its control are met within constraints set by the perceptual demands of two other key tasks: the control of bill (or feet) position, and the detection of food items/predators. Control of bill position, or of the feet when used in foraging, and timing of their arrival at a target, are based upon information derived from the optic flow-field in the binocular region that encompasses the bill. Flow-fields use information extracted from close to the bird using vision of relatively low spatial resolution. The detection of food items and predators is based upon information detected at a greater distance and depends upon regions in the retina with relatively high spatial resolution. The tasks of detecting predators and of placing the bill (or feet) accurately, make contradictory demands upon vision and these have resulted in trade-offs in the form of visual fields and in the topography of retinal regions in which spatial resolution is enhanced, indicated by foveas, areas, and high ganglion cell densities. The informational function of binocular vision in birds does not lie in binocularity *per se* (i.e., two eyes receiving slightly different information simultaneously about the same objects) but in the contralateral projection of the visual field of each eye. This ensures that each eye receives information from a symmetrically expanding optic flow-field centered close to the direction of the bill, and from this the crucial information of direction of travel and time-to-contact can be extracted, almost instantaneously. Interspecific comparisons of visual fields between closely related species have shown that small differences in foraging techniques can give rise to different perceptual challenges and these have resulted in differences in visual fields even within the same genus. This suggests that vision is subject to continuing and relatively rapid natural selection based upon individual differences in the structure of the optical system, retinal topography, and eye position in the skull. From a sensory ecology perspective a bird is best characterized as “a bill guided by an eye” and that control of flight is achieved within constraints on visual capacity dictated primarily by the demands of foraging and bill control.

## Introduction

“A bird is a wing guided by an eye” is a phrase that seems to capture the essence of modern birds. The phrase was coined by Rochon-Duvigneaud (1943) and has often been repeated. It elegantly defines birds from two perspectives; that their key behavior is flight and that this is guided by information extracted from the environment by vision. The implicit assumption is that the gaining of information to control flight had been, and still is, the key driver of avian visual capacities.

It is clear, however, that while birds may be highly dependent upon vision, information from other senses are important for the control of a wide range of behaviors, and that vision is used to control many behaviors beyond flight (Martin, 2014, 2017). When discussing the evolutionary pathway that led to the optical design of modern vertebrate eyes Nilsson (2009) argued that the changes to cameras eyes as they first evolved were neither continuous nor incremental. Nilsson argued that the evolution of eyes had been subject to periods of rapid change, as new visually guided tasks were hit upon through natural selection, followed by relative stasis. To capture this idea Nilsson suggested that the evolution of eyes has been the subject of “task-punctuated evolution,” in which there were longer periods of stasis alternating with shorter periods during which rapid structural and physiological changes occurred. This raises the important question of which tasks have in fact driven and continue to drive the evolution of bird eyes? Are the structures and capacities of birds' eyes primarily the results of the perceptual demands of flight, as Rochon-Duvigneaud suggested, or are other tasks key to understanding the functions of vision in birds? Is the control of flight, in actual fact, achieved within visual parameters driven by the demands of other tasks?

Nilsson and Pelger (1994) argued that the main features of vertebrate eyes had been arrived at through the process of punctuated evolution over a relatively short period of time. A conservative estimate suggested that a fully functional camera eye could have evolved in as little as 400,000 generations. However, vertebrate eyes have become increasingly differentiated and specialized in many and subtle ways. These are exemplified by the interspecific differences found in readily measured visual capacities, such as resolution, sensitivity, visual fields, and the topographical patterns of image analysis within retinas.

Natural selection driving such interspecific differences may have occurred more-or-less continuously and, indeed, subtle differences have been noted between closely related species suggesting recent evolutionary change. For example, the important functional differences in the visual fields of closely related ducks, shorebirds and ibises (Guillemain et al., 2002; Martin and Piersma, 2009; Martin and Portugal, 2011), and the differences in retinal topography in the eyes of closely related passerines (Lisney et al., 2012a; Coimbra et al., 2014, 2015; Moore et al., 2015, 2017), parrots (Mitkus et al., 2014) and Procellariiform seabirds (Mitkus et al., 2015), must have evolved relatively recently.

These subtle interspecific differences in vision have been interpreted primarily in the context of the foraging tasks that these birds conduct rather than differences in their flight capabilities and other behaviors, and so this raises the question of the importance of flight as a driver of bird vision. However, it should be noted that foraging behavior is better described than flight behavior in the majority of bird species. If vision is driven primarily by foraging, which aspects of that behavior are the key tasks and do they have to be traded-off against the perceptual demands of other tasks?

**Figure 1:**
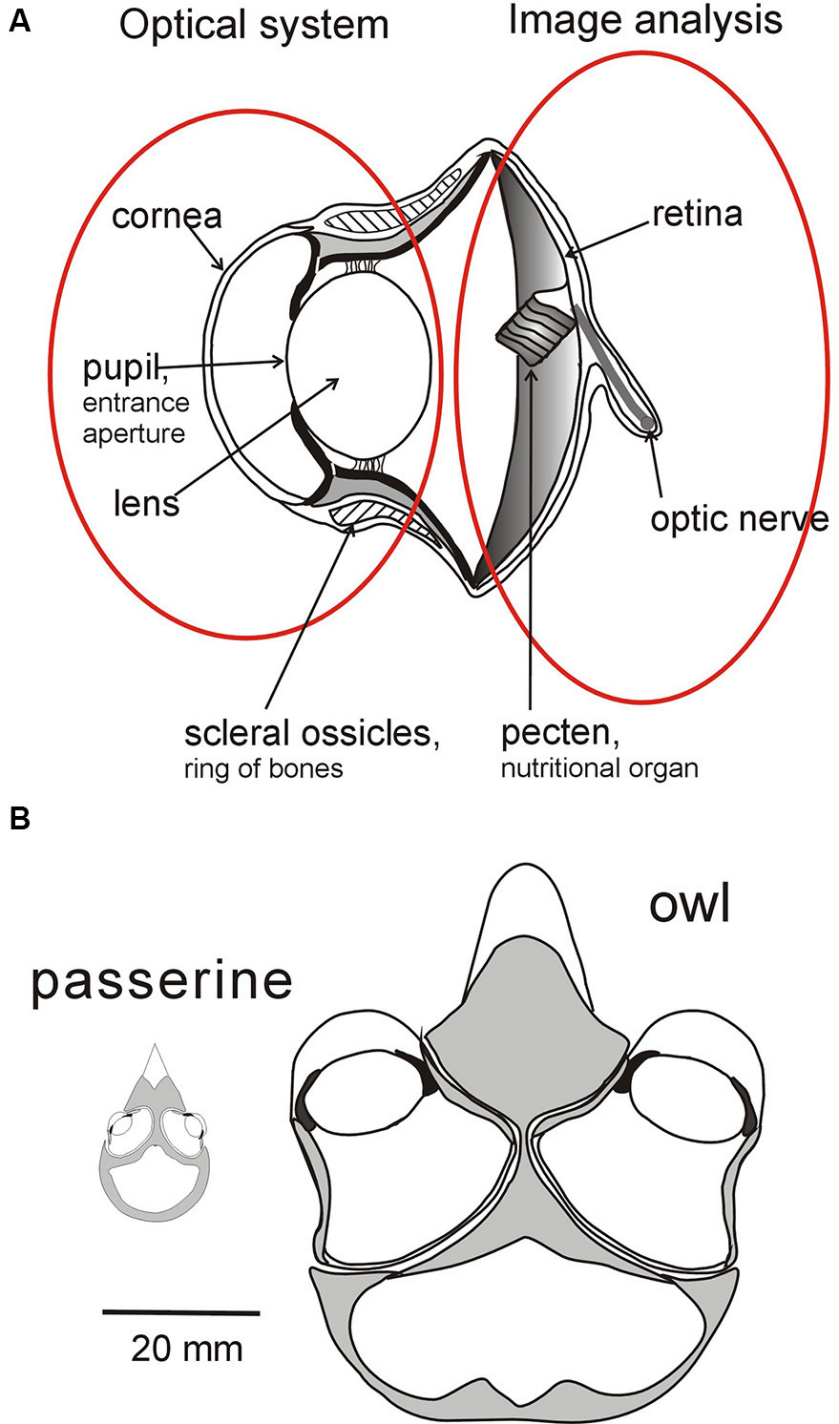
Sources of variation in bird eyes. **(A)** Diagrammatic cross section through the eye of an owl highlighting the optical system (lens and cornea) which projects an image of the world onto the retina which is the first stage of the image analyzing system. The characteristics of the two systems can evolve independently of each other within broad parameters. The optical system determines the brightness, size, and optical quality of the image, while variation in the distribution of photoreceptors and ganglion cells across the retina determines the fundamental limits on the information that is extracted from different regions of the image (Figure 2). Although the optical and analyzing systems are linked together their essential characteristics can evolve independently. **(B)** Variation in the placement of eyes in the skull of birds depicted by diagrams of cross sections through the head of an owl and of a small passerine. While eyes differ in size their placement in the skull and the extent of their visual fields are influenced not only by the size of the visual field of each eye's optical system but also by the ways in which the fields overlap to determine the extent of binocular field, the total field, and the width of the blind area to the rear of the head. See Figure 4 for a diagram of how the fields of the two eyes can be combined to give different total visual field configurations, and Figures 3, 5 for examples of different visual field configurations in birds. From Martin (2017).

**Figure 2:**
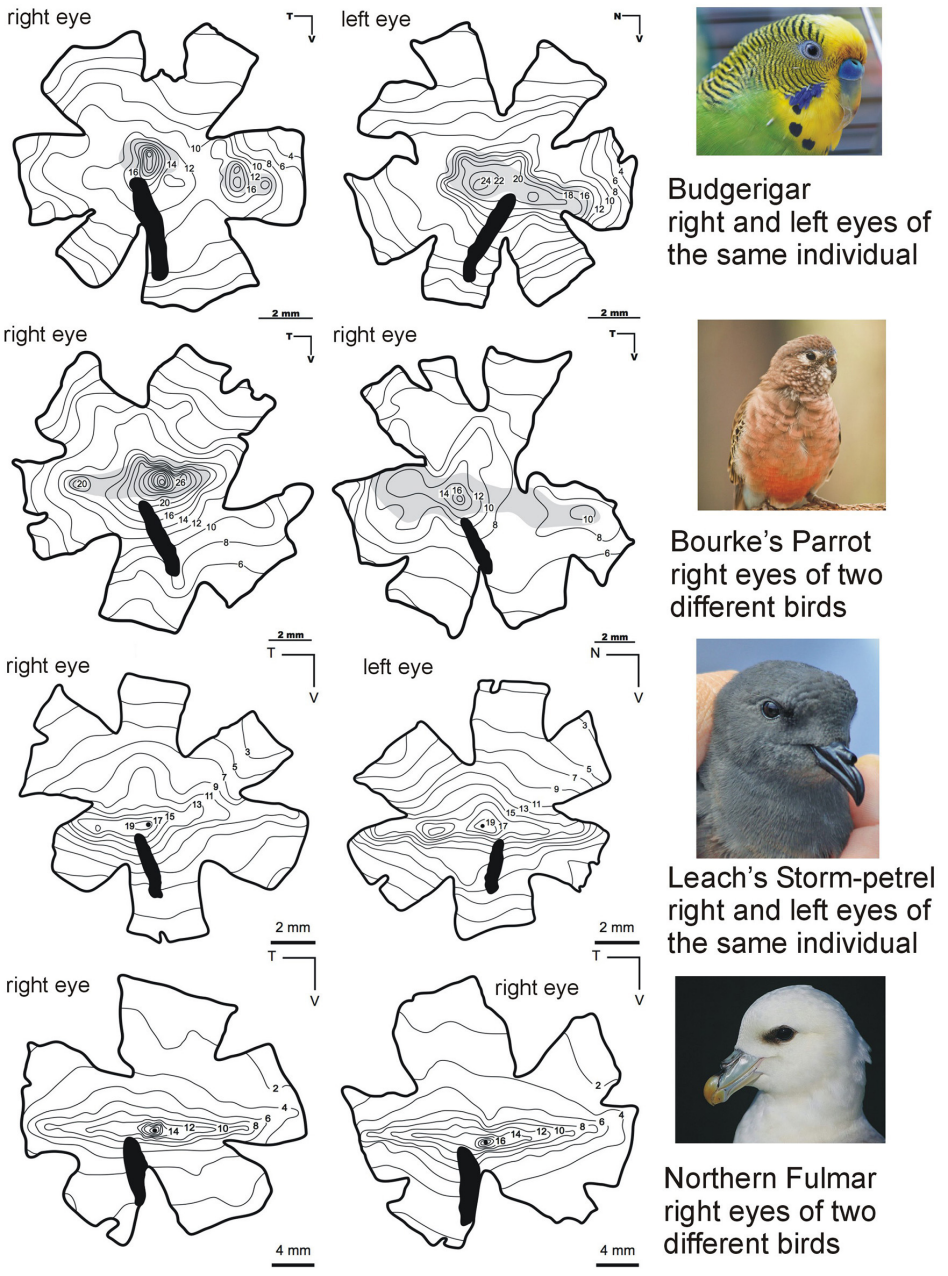
Complexity within the structure of the avian retina as depicted by patterns of ganglion cell density. Patterns can vary markedly between species and also between the left and right eyes within the same individual bird and this is evidence for the fine tuning of initial image analysis between and within species. This series of pairs of ganglion cells isodensity maps show examples taken from a range of birds which differ in their phylogeny and ecology. Marked differences can be seen between the two seabirds (Order Procellariiformes) and the two parrot species (Order Psittaciformes). The seabirds have distinct linear bands of higher density cells running approximately horizontally across the retina and these project toward the horizon when the birds are in flight. There are also regions of high density in the central part of the retina which project laterally. Significantly higher cells densities are found in the retinas of the two parrots compared with the seabirds. Also rather than a linear band of higher density there are two distinct regions of high density in each retina. Note, however, that the two examples of right eye retinas from Bourke's parrots show markedly different densities and the left and right eyes of the same Budgerigar seems to show considerable differences. Both parrots have a region of high density in the center of their retinas which therefore projects laterally. In the right eye of the Budgerigar there is a second region of high density in the nasal retina and this must projects backwards in the field of view, but this distinct region is absent from the left eye of the same bird. All diagrams from Mindaugus Mitkus, Lund Vision Group, Sweden. Photos credits: Budgerigar (Michael Cole), Bourke's Parrot (Daniela Parra), Leach's Storm Petrel (Fanter Lane), Northern Fulmar (Steve Garvie). From Martin (2017).

It is now established that in birds significant changes in key structures associated with foraging can occur at very short time scales. Driven by differences in foraging opportunities, it has been shown that bill structure can change over just decades (Grant and Grant, 2014). Whether such rapid evolutionary change occurs in sensory structures and capacities is not established, but it is a possibility. This is because the structures that underpin sensory organs are inherently flexible and give rise to individual differences in all aspects of sensory capacity and so provide the variability upon which natural selection can act. It is clear that the camera eye's optical system, image analyzing system, and the way that eyes are combined in the head, can vary independently of each other, and that these are at the root of differences in the visual capacities of bird species and of individuals (Martin, 2017; Figures 1, 2). This review draws together various strands of that information and uses it to focus upon the control of the bill (and feet) in foraging and how these are traded-off against the demands for the detection of predators.

## Which tasks drive the evolution of the sensory capacities of birds?

Resolving the question of what drives avian senses is somewhat problematic because it must rely upon a diverse suite of information that has to be carefully brought together to make an argument that should be more than a “just so story” (i.e., “a speculative style of argument that records anatomy and ecology and then tries to construct historical or adaptive explanations” Gould, 1985), and be built upon comparative studies that produce quantitative data and if possible cover a sufficiently wide taxonomic sample that statistical techniques can be employed to control for the confounding effects of phylogenetic relatedness. Although, as noted above, functionally significant differences in vision can occur between closely related (congeneric) species and so finding a firm basis for making comparisons may be often be difficult in the absence of data from a large sample of species. Nevertheless it is a worthwhile exercise since understanding what drives the sensory capacities of birds should lead to better understanding of how information is used generally in the control of bird behaviors and how it imposes limits on behaviors. Hence understanding what drives vision in birds should have applied value, particularly for understanding why birds often have fatal interactions with a variety of large and apparently obvious human artifacts (Martin, 2011, 2014).

For any comparative studies of eye structure and function there is a need to draw study species from a well-established phylogeny, from species which occupy different habitats, and from species which present a variety of behavioral repertoires. A sample which draws on these criteria is likely to include animals whose visual systems have been shaped by a range of different perceptual challenges and hence features which have evolved in response to different challenges may become evident. Fortunately, the approximately 10,000 species of extant birds (Gill and Donsker, 2017) provide a good taxon for such studies since on the whole their ecology and behaviors tend to be known in broad terms, and for many species there have been detailed behavioral and ecological studies (Gill, 2007). The taxonomy of birds is well established at the level of the family (Gill and Donsker, 2017). However, at higher taxonomic levels there is debate based around different technique of classification (Livezey and Zusi, 2007; Hackett et al., 2008; Jetz et al., 2012; Jarvis et al., 2014; Prum et al., 2015) and so deeper evolutionary affinities are not always certain.

## Key tasks

Among birds, and perhaps all animals, the tasks which have high informational demands, and are subject to strong natural selection on a daily basis, are locomotion, foraging, and the detection of predators (Martin, 2014). Less frequent but highly selected behaviors, which are also likely to have important informational demands, involve reproduction and the care of young.

The question is, are any of these key or primary drivers of sensory capacity? That is, are the informational demands of some tasks primary, while the informational demands of other tasks met within parameters set by the primary demands?

The discussion that follows argues that this is indeed the case and concludes with the proposal that the primary driver of sensory capacity in birds is foraging. Furthermore, because of the unique use of the bill in the foraging of the majority of birds, the key driver can be further refined to the quite specific informational demands for the control of bill position and the timing of the bill's arrival at a target. In some predatory birds the feet play the key role in prey capture and in these species the control of feet position when taking prey may make the same demands as the control of bill position in the majority of species (see for example, the analysis of feet position within the visual field prior to prey capture in an eagle, Martin and Katzir, 1999). The second most important task that drives vision in birds is the detection of predators. It seems, however, that accurate bill (or feet) positioning and predator detection usually make antagonistic informational demands with one requiring detailed information from the frontal field of view and the other detailed information from the lateral or posterior field of view. The result of this antagonism is that there is a trade-off between these two demands (Martin, 2014). However, getting the bill or feet to the right place and at the right time takes precedence over predator detection. This is perhaps because predator detection can usually be enhanced by behavioral adaptations involving scanning, the use of senses additional to vision, and social behaviors. However, accurate bill (or feet) position and timing can only be achieved using visual information available to the individual. The outcome of these trade-offs between the control of bill or feet position and detecting predators depends upon details of the foraging ecology of each species with the result that there are many subtle variations between species (Martin, 2014). The final conclusion of this review is that all other behaviors, including flight and reproduction, are conducted within the constraints set by the sensory information that is necessary to guide bill or feet position, and the requirements of predator detection.

## Key tasks and perceptual challenges faced by birds

### Flight

Whatever, the exact origins of birds (Chiappe, 2006; Zheng et al., 2013) it does seem to be clear that the control of flight was a task that the very first birds must have accomplished and so it has been assumed that even among the earliest birds flight may have required a high degree of specialization of visual systems to provide information that is both spatially accurate and processed at high speed (Alonso et al., 2004). Both attributes are thought necessary in order to cope with the demands of traveling at relatively high speeds and flightless birds almost certainly had ancestors that flew (Bunce et al., 2009; Phillips et al., 2010). It would seem reasonable, therefore, to suppose that the gathering of information necessary for the control of flight is likely to have been important throughout the evolution of the sensory systems of birds. However, many birds fly without the benefit of fine spatial information, there being a 30-fold difference recorded in the highest spatial resolution among diurnally active bird species and this becomes nearly 40-fold if nocturnal species are included (Martin, 2017). Furthermore, it seems highly likely that the highest spatial resolution may have evolved, as exemplified by eagles and vultures, for the detection of prey items and other foraging birds at great distance, rather than to perceive fine detail close by Martin (2017). Indeed eagles and old world vultures, which have the highest known spatial resolution of any vertebrate eye (Land and Nilsson, 2012), do not frequent spatially complex habitats, and their key informational demand when foraging is probably the detection of large food items at considerable distance, not at close range.

### Foraging

The task of detecting and procuring food is likely to pose a constant perceptual challenge in the majority, if not all, bird species. A key overall constraint on birds throughout their evolution has been the requirement to combine low body weight with high power output (King and King, 1980), with the result that many bird species have evolved to forage very frequently, almost continuously, during their active episodes every day. Furthermore, this foraging is usually for a narrow range of food types or individual items (Gill, 2007). Present day bird species forage for a very broad diversity of food items and the effective initial detection of each type of food item pose specific perceptual challenges. The types of food items utilized by different species of bird species vary from the relatively large to the minute, and from evasive and highly mobile prey items, to fixed or sedentary foods. Among items found in the diets of birds are animals of many faunal types, including forms that fly, live buried beneath surfaces, live on terrestrial surfaces, or in water (Gill, 2007).

Different diets are associated with specialized methods of obtaining food nearly all of which involve using the bill as the sole tool. Probing, pecking, lunging, aerial pursuit, excavation of substrates including soils and wood, pursuit of prey beneath a water surface, filtering items from water and mud, grazing, trawling water and the air; all of these foraging methods pose a diverse variety of perceptual challenges. Furthermore, these tasks must be dealt with frequently, in some species almost continuously, by a bird throughout its waking hours. Extracting the required information from the environment that allow birds to forage in these specialized ways are likely to be the result of rigorous natural selection.

Rigorous selection of this kind is likely to have comparable outcomes to the exacting natural selection which can result in rapid changes in the structures that birds use to procure individual items of food, particularly the size and shapes of bills. The efficient acquisition and manipulation of food items can require such subtle structural changes to bill shape and size that they can evolve “in real time” (Weiner, 1994; Grant and Grant, 2002, 2014). However, having the right bill shape and size is of little value if it cannot be targeted, or the timing of bill opening controlled with accuracy and precision. With the exception of those small number of species that can feed by filtering substrates (e.g., some ducks, some procellariiforms, flamingos) or by trawling insects from the air (e.g., nightjars and swifts), the tasks of timing and controlling the bill's position in foraging always needs to be done highly accurately and precisely. Such tasks have to be achieved every time a food item is ingested. The need for such accuracy is likely to be equally acute in all birds that employ vision to locate and take food items. Many birds which feed on immobile objects, such a seeds and fruits, and birds which feed upon insects sitting on surfaces, will need to control the timing and positioning of the bill almost continuously throughout their waking life. However, if the foraging task is done less frequently it may be even more exacting since less frequent feeders are likely to be taking larger but mobile and evasive prey. To feed in this way, accuracy of bill position and the timing of its arrival at the food object are also paramount because there is often only a single opportunity to take a particular item, otherwise it escapes.

### Predator detection

A key task that is faced by perhaps all bird species is avoiding being detected and consumed by a predator. Detecting a potential predator is one that faces most bird species more-or-less constantly whenever they are active (Sansom et al., 2009; Cresswell, 2011; Fernandez-Juricic et al., 2011a; Tyrrell and Fernandez-Juricic, 2015). It has been argued that the task of detecting predators using vision has been a key feature of animal life since the Cambrian Explosion 540 million years ago (Parker, 2003) and no habitats are predator free for long. For example, on the islands of New Zealand, which are notable for having been without mammalian predators for 80 million years until they were introduced by humans in recent times, avian predators were always present (Worthy and Holdaway, 2002; Wilson, 2004), and on recently formed volcanic islands, such as the Hawaiian and Galápagos groups, predator-prey relationships among birds were soon established.

### Reproduction

The tasks of reproduction have been shown subject to demanding selective pressures (Davies et al., 2012). Reproducing can take up a large proportion of an individual bird's life time although the actual amount of any one day devoted to tasks specifically serving reproduction, as opposed to maintenance and provisioning of self or young, may be relatively small. Some fascinating aspects of the behavior of birds often involve postures to display particular plumage used as signals during reproductive behavior and their detection clearly has an important informational component. Investigation of such displays and related plumages have been discussed within a sensory ecology framework, for example, see the work of Endler and Mielke (2005), Endler et al. (2005), and Hagelin (2007).

The information that underpins these reproductive behaviors have often been studied in detail. However, because reproduction occurs in discrete episodes in the life of an individual, selection based upon its informational demands will be intermittent compared with the more continuous selection that is likely to result from the execution of the daily tasks of predator detection, foraging, and locomotion. It can be hypothesized that the particular informational requirements of reproductive behavior are carried out within the context of the informational demands of these more ubiquitous behaviors. Furthermore, in the majority of bird species, behaviors that are associated specifically with reproduction involve the use of the bill as a tool for the gathering of nest material and in nest construction. In many species placement of the bill when feeding young must also be done with accurate positioning and timing. These are the same kinds of demands that apply in foraging and, of course, predator detection will be a constant demand in all phases of reproduction.

## Competing tasks and competing information

It is highly probable that the perceptual challenges posed by predator detection, foraging and locomotion, apply more-or-less constantly in the lives of most birds. Furthermore, it is likely that they have applied throughout the evolution of birds. Most birds face the potential risk of predation on a continuous basis. Often, the perceptual challenges associated with exposure to predation, foraging and simply moving about, will occur simultaneously. In some instances, however, there may be switching between theses perceptual challenges and the retrieval of information for different tasks may actually compete with each other. A foraging bird, for example, could need information to guide its detection and seizing of a food item but simultaneously it will need to gain information on the possible presence of predators. Such apparent competition between tasks and the information necessary for their execution have been studied in some detail, for example by studying how foraging birds behave when a predator is introduced under controlled conditions (Devereux et al., 2006; Fernandez-Juricic et al., 2008).

The information necessary to answer such different but frequent challenges may be quite different. In one particular species, for example, gaining information specifically for the detection and intake of food may be antagonistic to the requirements for information necessary for predator detection. In fact it has sometimes been argued that because the informational demands of the tasks of predator detection and foraging are so different, they are not conducted simultaneously and switching between discrete behaviors is required. For example, breaking off foraging with the head down and lifting or reorienting the head to scan for predators (Guillemain et al., 2002; Fernández-Juricic et al., 2004; Fernandez-Juricic et al., 2008; van den Hout and Martin, 2011; Martin, 2012).

Compromises and/or trade-offs can occur within a single sensory modality, especially in vision (Martin, 2017). The multifaceted nature of vision and its different “capacities” which are measured independently of each other, means that it is difficult to often understand the trade-offs and compromises that have occurred, although it is possible to understand that different facets of visual performance cannot all be maximized simultaneously (Land and Nilsson, 2012).

## Visual fields as an exemplar for the investigation of what drives vision

Visual fields provide a good comparative base from which to understand how the different perceptual challenges presented by flight, foraging and predator detection, have been traded-off within avian visual systems and how this has resulted in the fine tuning of vision to different perceptual challenges. Such tuning has resulted in functionally significant differences in visual ecology even between congeneric species (Guillemain et al., 2002; Martin and Portugal, 2011). Data on visual fields in birds are available from studies using the same technique in over 60 species (from 31 families and 20 avian orders) and have been summarized in Martin (2017).

Similar arguments can be made with reference to other properties of bird vision especially the patterns of receptor and ganglion cell distributions (which may take the form of approximately linear or circular areas), and the numbers and positions of foveas within the retina. Although the functions of these patterns are yet to be reviewed in detail, it has long been argued that they are the product of different perceptual challenges arising from the conduct of particular tasks in different environments (Wood, 1917; Walls, 1942; Hughes, 1977; Fernandez-Juricic et al., 2011b). This is most readily exemplified by interspecific differences in the number of foveas and areas, and their positions within the retina (Figure 2), and these differences are typically explained by reference to the detection of particular targets, especially prey items and predators, in particular sectors of the visual fields (Galifret, 1968; Dolan and Fernandez-Juricic, 2010; Lisney et al., 2012b, 2013; Mitkus et al., 2014; Coimbra et al., 2015; Mitkus, 2015; Moore et al., 2017).

### General characteristics of the visual fields of birds

**Figure 3:**
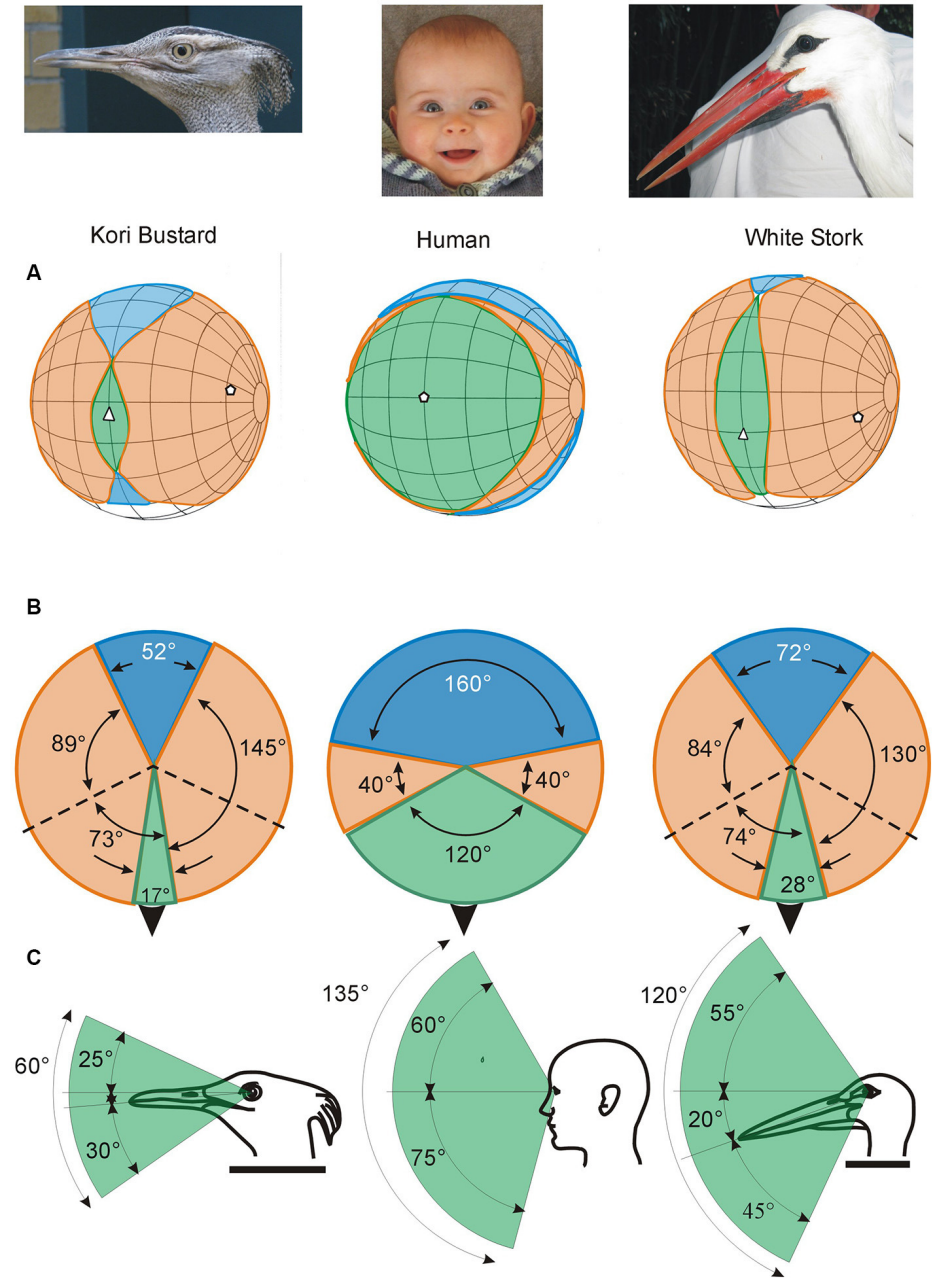
Variation in the visual fields of birds and how they differ from the visual field of humans. The ways in which the visual fields of each eye are brought together to form the total visual field of an animal can vary markedly (Figures 1, 4), producing significant differences in the size (width and vertical extent) of the binocular field (which is where the fields of the two eyes overlap, indicated in green), the blind area behind and above the head (indicated in blue), and the portion of space that is viewed by each eye alone (indicated in orange). Shown here are the visual field characteristics of two birds, Kori Bustard *Ardeotis kori* and White Stork *Ciconia ciconia*, and the visual field in humans. The top row of diagrams **(A)** show the visual fields as projected onto the surface of a sphere surrounding the head. The grid (at 20° intervals) follows, convention latitude and longitude but with the equator aligned vertically in line with the media sagittal plane of the skull. The projections of the bills, and in humans the nose, are indicated by the white triangles. The middle row **(B)** shows schematic horizontal sections through the visual fields with the black arrow indicating the direction of the bills. The bottom row **(C)** presents vertical slices through the visual fields in the median sagittal plane of the head showing the vertical extent of the region of binocular overlap and its position relative to the bill. Very marked differences in these visual fields are apparent with the human differing dramatically from the two birds. However, the birds also show significant differences from each other, this is despite the similar size of the visual fields of the individual eyes in these two species (162° and 158° wide in the bustard and stork, respectively). Thus small differences in the position of the eyes in the skull (Figure 1) have influenced all parameters of the visual fields in these two birds. Redrawn and modified from Martin (2011). From Martin (2017).

From a human perspective we “know” that the world surrounds us. However, at any one moment that is not how we experience it. Humans experience the visual world as “in front” and we seem to constantly move forwards, into it. This is a result of the particular configuration of our visual field, it is the “human eye view” (Figure 3). Visual fields define the space around the head of an animal from which information can be extracted at any one instant. Human eyes are placed in the front of the skull; basically our eyes project horizontally, neither up nor down, not sideways or back, just forwards. Furthermore, what the left eye sees is very similar to what the right eye sees, that is, we have a large area of binocular overlap, with each eye looking at the same scene from a slightly different viewpoint. The whole of the human visual field lies within the hemisphere in front of the face. However, compared with most vertebrates, including all birds, human eye placement, and our resultant visual field is unusual (Figure 3). Birds are, in effect, surrounded by their visual world and they “flow through” it, rather than move into it (Martin, 2012, 2014). As a bird moves through the world an object can be tracked from directly in front to the rear of the head.

**Figure 4:**
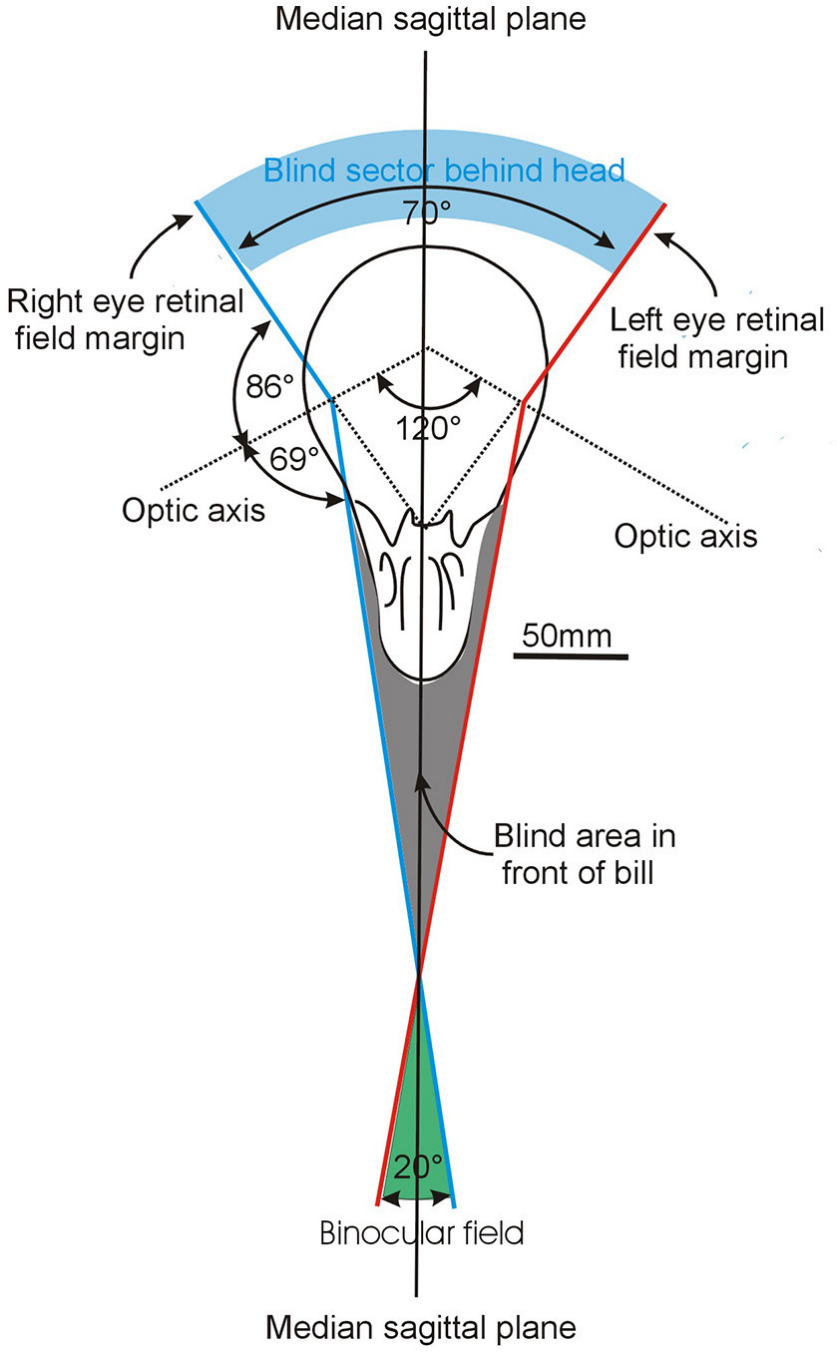
How the visual fields are brought together to produce the overall visual field. The example shown here is based on analysis of Common Ostriches *Struthio camelus* and while the elements are the same in all bird species, the size of the key components differ between species as a result of differences in the size of the field of each eye and the way they are brought together in the skull (Figure 1) The diagram shows a section through the skull in an approximately horizontal plane with the margins of the left and right eye visual fields picked out in red and blue, respectively. Each eye has a field of 155° and they are brought together to achieve a binocular overlap of about 20° in the horizontal plane i.e., each eye projects contralaterally by 10° across the median sagittal plane of the head (the plane that bisects the head into two mirror image halves). Modified from Martin (2009).

In birds the eyes are on the side of the skull, each eye looks outwards at a different scene and the overlap in each eye's visual field is relatively small, typically between 20° and 30°, in some species as wide as 60°, but it can be as narrow as 5°-10° in some birds (Figures 3, 4).

**Figure 5:**
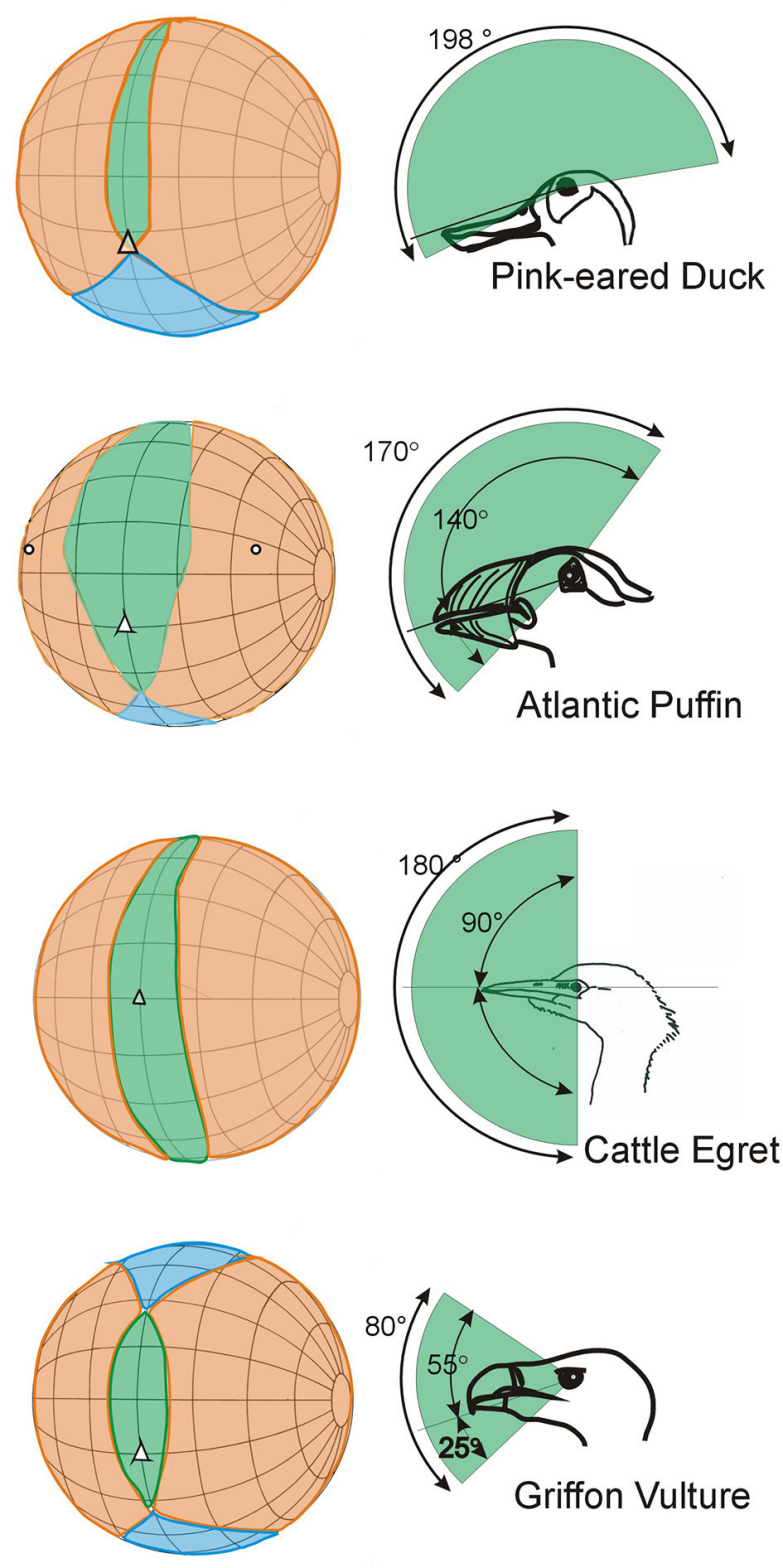
Further examples of variation in the visual fields of birds with an emphasis upon differences that result from the way that the optic axes of the eyes project with respect to the horizontal plane. The conventions in these diagrams follow those used in Figure 3. The diagrams in the left hand column show how the binocular regions can vary in width and position relative to the projection of the bill, and also in their vertical extent. The right hand column emphasizes these differences and shows the vertical extent of the binocular region and its position in the median sagittal plane of the head. In the duck, puffin and egret the binocular fields extend through approximately 180°. In the duck the eyes are positioned to give the bird comprehensive visual coverage of the world above it, but it cannot see below the level of its bill. In the egret the bird can see all of its frontal hemisphere, including its feet, but has a blind area behind the head. The puffin has extensive visual coverage centered obliquely upwards. The vulture has a much small binocular region and an extensive blind area above, below, and behind the head. From Martin (2017).

In no birds do the eyes look directly forwards. In many species the eyes are directed laterally and are also positioned relatively high in the skull with the optical axes projecting slightly dorsally, not horizontal. For the large majority of bird species their visual world is in effect all around; there is a very small blind region above and to the rear of the head and in some species there are no blind areas above the horizontal (Figure 5). There are even bird species among the shore birds (Scolopacidae) and ducks (Anatidae) that have comprehensive visual coverage of the hemisphere above the horizontal (e.g., Figure 5, Pink-eared Duck), and also extensive coverage to the sides and front below the horizontal. This means that instantly these birds can extract visual information from the complete volume that surrounds them, except from the space occupied by their own body. In other birds the eye axes point slightly upwards (e.g., Figure 5, Atlantic Puffin), in some they point slightly downwards. This means that they are able to examine objects at their feet when the bill is held horizontal (e.g., Figure 5, Cattle Egret), or they are able to comprehensively scan below when foraging on the wing (e.g., Figure 5, Griffon Vulture).

## Functional interpretations of the visual fields of birds

The argument presented here is that both the general and detailed features of bird visual fields, down to the level of individual species, have been driven primarily by the informational challenges associated with foraging. The key challenge in this is the accurate positioning of the bill (in some species the feet/talons) when taking food items, but this must be simultaneously traded-off against the informational demands of predator detection. In certain species there is an additional challenge; the need to avoid imaging the sun upon the retina. It is argued below that the perceptual requirements for the control of locomotion are not a key challenge but are met within the perceptual requirements for efficient foraging and predator detection (Martin, 2014).

### The key functions of bird visual fields

A number of strands of evidence support the idea that controlling bill position, including the accurate timing of its arrival at a target, is the most demanding task that vision is used for by birds. The second most demanding task is the detection of predators. These tasks, however, make competing demands and the configurations of visual fields are primarily the result of this competition. The strands of evidence in support of this are summarized in the next paragraph and then discussed in more detail.

The foraging of most birds requires exact positioning of the bill (or in some species the feet) with respect to a target, regardless of whether the items are taken by pecking or lunging. Control of bill position (both the direction of travel toward the target and time to contact the target) can be achieved from the optic flow-field produced as the head (to which the bill is rigidly attached) moves toward the target. Optic flow describes the way in which the image of the world moves across the retina as the head moves through space. It is regarded as a foundation of perception in both vertebrates and insects (Lee, 1980; Srinivasan, 1996) and its fundamental role in the control of various aspects of flight behavior, especially timing of approach to a target in birds have been established (Lee et al., 1991; Davies and Green, 1994; Bhagavatula et al., 2011). Birds typically detect targets visually in their lateral fields of view, probably employing a region in each eye which combines the highest quality optics (usually along the optic axis) and the retinal region specialized to provide high spatial resolution (Martin, 2009). Visual control toward the target is only subsequently passed to the frontal portion of the visual field, which is the sector in which the bill direction projects. This may occur, however, only at a close distance from, and a short time before, contact with a target. This facilitates the bill to be accurately directed toward a target and of equal importance to time the arrival at the target with accuracy. This is essential so that bill opening (or the spreading of talons by a predatory species) can be precisely co-ordinated with arrival at the target and the object grasped. The pecking of some birds has a ballistic phase in which the eye lids are shut during final approach to a target. Prey may be taken in the feet by some birds and these are swung up before the head, into the region of the binocular field, just prior to prey capture (Martin and Katzir, 1999).

## Panoramic vision

Complete visual coverage of the hemisphere above and around the head is found in a small number of bird species. Such panoramic vision is achieved with a narrow sector of binocular overlap (<10°) which extends through 180° from directly in front of the head to directly behind it (e.g., Figure 5, Pink-eared duck). Having complete visual coverage of the world all around the head would seem to be the ultimate adaptation to the demands of predator detection. Given its potentially great utility, panoramic vision might be expected to be relatively common among bird species. It is, however, found only in a small number of species. While many birds have extensive visual fields most have a blind area behind the head leaving them more vulnerable to predator attack (Figure 3).

The presence of these blind areas and their absence in only certain species is evidence that controlling bill position and the detection of predators are tasks which have different informational demands that are in competition. Total panoramic vision appears to have evolved independently in just two bird orders which are distantly related (Jarvis et al., 2014); ducks (Anseriformes) and shorebirds (Charadriiformes). Only a few species in these taxa have totally panoramic vision, but those that do, share a common feature in that their foraging does not require visual control of bill position; foraging relies upon tactile information derived from bill-tip organs (Martin, 2017). Accurate visual control of bill position seems to place an important constraint of visual field configuration, but when that constraint is removed, it appears that natural selection has driven toward the evolution of comprehensive (panoramic) visual coverage above and around the head as an aid to predator detection. It is noteworthy that in these species, the width of binocular overlap is very small, between 10° and 5° even in the direction of forward flight (Figure 5). However, these birds are able to fly fast even in complex habitats. This suggests that a frontal binocular field of this narrow width is sufficient for the control of flight. Thus, it seems safe to conclude that the function of broader binocular fields is related to the control of bill position, not flight control.

## Differences in visual fields between closely related species

There is evidence from both ducks and shorebirds that the gaining of comprehensive vision can evolve relatively rapidly. This is indicated by the finding that there are significant differences in vigilance behavior in two ducks within the same genus, and that these differences are explained by differences in their visual fields. These differences in vigilance behavior have been observed between the non-visual (tactile and filter) foraging Northern Shovelers *Anas clypeata* and the visually guided foraging Eurasian Wigeons *A. Penelope* (Guillemain et al., 2002). Wigeons are selective grazers guided by visual cues and have a wider binocular field which embraces the projection of their bill tip, with the result that they have a blind region behind the head. On the other hand Shovelers have comprehensive visual coverage of the celestial hemisphere. Thus, these congeneric species, which can be observed exploiting different resources in the same locality, differ in their visual field configurations, foraging technique, and also in their vigilance behavior. This demonstrates that subtle, but behaviorally significant, differences in visual ecology can occur between closely related species. A more recent comparative analysis of binocular field characteristics and estimates of visual acuity in buntings and American sparrows (Emebrizidae) also showed subtle but functionally significant differences between closely related species. As in the ducks these differences could be related to differences in foraging and vigilance behavior (Moore et al., 2015).

## The perceptual demands of bill control vs. predator detection

The above examples indicate that closely related bird species, which employ different perceptual cues (visual or tactile) for foraging, can differ in their visual field characteristics and that these differences are functionally important. This suggests that visual fields (plus the anatomical and optical structures which underpin them) are driven primarily by the informational demands of foraging, although similar studies on a wider range of species are necessary to adequately test this hypothesis. Such evolutionary outcomes regarding the informational demands for the visual control of bill position and timing may be analogous to the more well-studied subtle variations in bill form that are driven by the mechanical demands of foraging (Grant and Grant, 2002).

These examples reinforce the hypothesis that the configuration of visual fields are driven primarily by the informational challenges of foraging which are traded-off against the requirement for predator detection. It is argued that, “Only in those species which do not need to use vision to guide their bill position during foraging, is comprehensive visual coverage of the world about the bird attained” (Martin, 2014). Not requiring visual cues to guide foraging is, however, not sufficient to lead to the evolution of comprehensive vision. It is also necessary that the bill does not require fine visual control for any task, not just foraging. Thus comprehensive vision is, in fact, found only among birds which also do not need to position their bills accurately for two other key tasks; nest construction and the provisioning of young. Both the ducks and shorebirds use simple nests which do not require elaborate construction, and their young are precocial. That is their young hatch in an advanced stage of development and self-feed from hatching. They are never provisioned by their parents; parental care is limited to brooding and protection from predators. Most other birds must use their bills for foraging, for nest building, and for the provisioning of young, all tasks which require accurate position and timing of the bill.

A telling example that makes this clear is provided by flamingos (Phoenicopteridae) (Martin et al., 2005). They are filter feeders, having highly specialized structures within their bills to remove minute resources from filtered water and mud, yet unlike the filter feeding ducks they do not have comprehensive vision. The reasons for this seems to be that they build nests which are a shaped mound of mud constructed with the bill, and, crucially, their young have to be fed very accurately by “crop milk” (a secretion from the esophagus) which is dripped into their open mouths. Thus, despite their filter feeding, flamingos require vision that allows accurate bill placement so that young can be provisioned. This results in a relatively broad binocular field into which the bill projection falls, and a blind area behind their head.

## The function of binocular vision in birds

Stereopsis and the perception of relative depth have become regarded as the prime function of binocular vision in humans and other primates, and it has often been assumed that these same functions apply to all instances of binocular vision. However, it seems unlikely that this is the case among birds.

With the exception of evidence from Western Barn Owls *Tyto alba* (Pettigrew and Konishi, 1976; Pettigrew, 1979; van der Willigen et al., 1998, 2003), there is only limited evidence that binocularity in birds is associated with stereopsis, a higher order visual function that results in the perception of solidity and relative depth extracted from the disparity between each eye's view of the same objects (Martin, 2009). An earlier study demonstrating stereopsis in American Kestrels *Falco sparverius* (Fox et al., 1977) has not been replicated and may have been subject to artifacts caused by the flickering nature of the displays used (McFadden, 1994). There is evidence of binocularly derived relative depth information in Rock Doves *Columba livia* that is based upon convergence eye movements and accommodation cues. However evidence of retinal disparity neurons of the kind thought to underlie stereopsis in owls and mammals (Barlow et al., 1967) have not been found in Doves. Binocularly driven neurons have been sought in doves. However, those that have been found have fields that project approximately180° apart and are thought to be involved in the control of turning movements, rather than the perception of relative depth (Frost et al., 1983; Wylie and Frost, 1990).

It has been argued (Martin, 2009) that binocularity in birds is, in fact, a consequence of the requirement for having a portion of the visual field that looks in the direction of travel of the head/bill. Hence each eye must have a contralateral projection, that is, each eye must look across the central plane of the head (Figure 4). It is true that this results in a region which is perceived by two eyes simultaneously and so it is labeled a binocular field. However, having two eyes extracting information from the same region is not the same as that region being analyzed with binocular vision in the sense in which it is usually understood in mammals i.e., regarded as synonymous with the process of stereopsis (Martin, 2009).

For any visual system the most vital information, more important than recognition of an object, is accurate determination of an object's position. Indeed it is argued that the main driver in the early evolution of vision systems was toward increasing accuracy in spatial resolution which meant increasingly accurate determination of the direction in which objects lay with respect to the viewer (Nilsson, 2009).

The next most important piece of information that vision provides is the time it will take to contact an object, that is, when will the object arrive at the observer or when will the observer arrive at the object? The actual identity of an object and its specific distance from a bird is likely to be of less importance than knowing the direction in which it lies, and crucially the length of time before contact will be made with it. This type of information is directly available from optic flow-fields (Lee and Lishman, 1977; Lee, 1980). It has been shown convincingly that birds use flow-field information to control apparently exacting tasks. For example, it has been shown that hummingbirds (Tochilidae) and Northern Gannets *Morus bassanus* when carrying out maneuver that require accurate visual information on object location and the time to reach it, employ flow-field information (Lee and Reddish, 1981; Lee et al., 1991) and there is increasing evidence that birds use flow-field information to guide flight (Bhagavatula et al., 2011) and landing (Lee et al., 1993). Optic flow-field information in mammals is processed in the accessory optic system (Giolli et al., 2006) and in the pretectum (Gamlin, 2006). A similar accessory system of the visual part of the brain has been identified in birds (McKenna and Wallman, 1985; Pakan and Wylie, 2006).

Information is potentially available from flow-fields that can be detected in any part of the retina. Information concerning time-to-contact a target and the direction of travel toward it are, however, extracted most efficiently when vision surrounds the target. This will result in an optical flow-field which expands symmetrically about the image of the target (Martin, 2009). This is the configuration that applies in the tasks described above as the key drivers of avian vision. When a bird is lunging or pecking at an object both its position and the time-to-contact need to be determined accurately. The crucial factors is that for a flow-field pattern to expand symmetrically about an object toward which the bill is directed, the visual field of each eye needs to extend across the median sagittal plane of the bird, that is, there must be contralateral vision (Figure 4) (Martin, 2009). Here, the important concept is contralateral vision, not binocular vision. It may be more appropriate to consider that binocular vision *per se* is the product of the requirement to have eyes that look forward across the median sagittal plane of the head. Such an arrangement means that movement toward a target by the bill produces a symmetrically expanding optic flow-field. Binocular vision in birds should therefore not be considered an adaptation that evolved to achieve simultaneous views of the same object from slightly different positions (which may be the case in mammalian species which have stereopsis). Rather binocularity may be driven by the requirement to place the bill, or the projection of its direction, at the center of a symmetrically expanding flow-field. What is important is contralateral vision rather than binocular vision as such.

It can be hypothesized that binocularity in birds functions to provide information on the direction of travel and time-to-contact a target. However, this information can be provided by each eye independently and for this reason it might be more appropriate to refer to “contralateral vision” rather than “binocular vision,” since the latter brings with it assumptions concerning the percept of solidity and stereopsis with which binocular vision in birds does not appear to be generally associated. Thus, in the majority of birds the function of binocularity would seem to lie in what each eye does independently rather than in what the two eyes might be able to do together.

## Conclusion: the key drivers of visual fields in birds

The arguments presented above support the hypothesis that two key tasks drive the configuration of visual fields in birds. The primary driver appears to be the perceptual challenges of foraging; specifically these are the control of bill (or feet) position, and timing their arrival at a target (Martin, 2014). This requires contralateral vision in the frontal field. The second driver appears to be the detection of predators and this requires vision over as wide a sector of space as possible around the head. Because each eye has a limited visual field (visual fields of a single eye is typically between 160° and about 180°, Martin, 2017), increased contralateral projection must result in a smaller total visual field and a blind sector behind the head. Thus, these two drivers make competing demands. They can be considered primary and secondary because only under the specific circumstance of bill position not having to be controlled by vision, does the requirement for predator detection result in comprehensive visual coverage.

There is a further important difference between the two key tasks that drive vision. The control of bill position requires information extracted from the world that lies in front of, and relatively close to, the bird. The detection of predators, on the other hand, requires information that lies laterally, or even to the rear of the bird's head, and is concerned with information from locations that are remote from the bird and it is this which probably drives higher spatial resolution in lateral fields.

The regions within the visual field where there is high spatial resolution, indicated by retinal regions of high photoreceptor and ganglion cell density (Tyrrell et al., 2013) (Fernandez-Juricic et al., 2011b), project laterally, not directly forwards. The lateral fields may be served by one or two foveas and/or a linear band of high density ganglion cells which align roughly with the horizon when the birds are at rest or in flight (Hughes, 1977; Meyer, 1977; Mitkus, 2015). Ever since such retinal topography was first described (Wood, 1917) these patterns have been correlated with the regions associated specifically with foraging or with the directions from which predators are most likely to attack. Such analyses are reinforced by more recent and detailed descriptions of retinal topography (Hughes, 1977; Fernandez-Juricic et al., 2011b; Lisney et al., 2012b, 2013; Mitkus, 2015) and there is no evidence that these patterns are associated with the control of flight. In some species e.g., Budgerigars a region of high spatial resolution appears to project into the posterior field of view (Figure 2), which is the direction from which predatory attack is more likely to occur (Mitkus et al., 2014).

There is evidence that predatory birds, such as Peregrine Falcons *Falco peregrinus* detect their prey at a distance using lateral vision, using the regions of high photoreceptor density which project laterally and slightly forward. When approaching prey Peregrines frequently do so along a curved path which keeps the prey approximately in the central field of view of a single eye and they pass control to the frontal binocular region just prior to prey capture (Tucker, 2000; Tucker et al., 2000). That is, the bird does not usually sight the prey into its binocular (frontal) field until just before prey capture. Thus distant prey is probably initially detected using lateral high resolution vision while the control of the bill and feet close to the time of prey capture probably employs frontal, lower resolution vision, and this comes into play only at close range. However, there is evidence that other falcon species may use frontal vision quite early on in the pursuit of prey and switch between the use of the difference foveas during a pursuit flight by turning the head (Kane and Zamani, 2014).

Such use of lateral vision for detecting food items with control passing to forward vision for final prey capture in the bill at close quarters has been reported in other species. For example, in terns foraging over mud flats for crabs (Land, 1999), thrushes searching on the ground for earth worms (Montgomerie and Weatherhead, 1997), and in domestic chicks when detecting grain from amongst grit (Rogers, 2008).

## A note on nocturnality

A reviewer of this paper argued that owls (and other nocturnal birds) are exceptions to the general argument presented above. However, no suggestions were made as to what the results of this exception might be. Why should owls and other nocturnally active birds such as, nightjars (Caprimulgidae) and kiwi (Apterygidae) be thought to be exceptions to the general thrust of the argument? What might be different about the demands of extracting information at lower light levels that would mean that the primary evolutionary driver of vision is not concerned with control of the positions and timing of the bill or feet toward a target but is rather concerned with the control of flight? It should be noted that not all owl species are nocturnal in the sense of completing all aspects of their life cycle between dusk and dawn. The sensory adaptations of nocturnal birds and their relationships to the challenges of general mobility and foraging are topics that have been addressed in detail a number of times in the past (Martin, 1986, 1990) and also recently, Martin (2017).

These reviews indicate that the frontal visual fields of owls show no special features compared with other raptors. Their binocular field is similar in width to those of passerines and is not the broadest recorded in birds. As stated above, the broadest binocular fields among birds are found in crows (Troscianko et al., 2012). The use of acoustic cues to guide owls to prey targets is well established but so also is visual guidance of the feet to take prey items when light levels are sufficiently high. Furthermore, the feet are raised just before prey strike to lie within the binocular field, suggesting that during prey capture, as in diurnal raptors, the feet may be guided by cues from the flow field within the binocular region (Martin and Katzir, 1999).

The visual fields of nightjars (Caprimulgidae) show high similarity to other non-passerine species. Although nightjars may trawl blindly for small insects they are also known to take larger individual insects in aerial pursuit and this is highly likely to be under visual control. Oilbirds *Steatornis caripensis* (now regarded as closely related to nightjars) are perhaps the most nocturnal of all flying birds. They roost and nests in caves by day and emerge to forage for fruit in the tree canopy at night. Their eyes are large and have the lowest f-number so far recorded in a terrestrial vertebrate and may also have an exceptionally sensitive retina (Martin et al., 2004b). However, their visual fields are very similar in general configuration to other non-passerine species (Martin et al., 2004a).

The visual fields of the flightless Kiwi are different to the majority of birds but Kiwi are dependent upon non-visual senses to guide their behavior and their vision shows evidence of regressive evolution (Martin et al., 2007). Kiwi do not appear to be visually guided in their foraging and, of course, they do not use visual cues for the guidance of flight. That the sensory systems and behavior of Kiwi are so different to other birds means that they cannot be considered as supporting or rejecting the central argument of this review.

It should also be noted that there are many instances of nocturnal behaviors in a wide range of bird species beyond the owls, nightjars, and kiwi. For example, many shorebirds, waterfowl, and diving birds forage at night and many passerine species routinely migrate at night. Many birds which seek their food underwater by diving to depths may forage at night, and some species, including penguins and auks, routinely forage at such depths that they can be regarded as nocturnal foragers even if they dive during the day. To go into detail on the vision and other senses in all such instances of nocturnal behavior in birds (or birds which may forage at low light levels) would take this review in a very different multisensory direction, but a reader interested in this should consider looking at Chapter 6 of Martin (2017).

Owls were referred to by the reviewer as an exception among birds although the exact basis of this exceptionality was not spelt out. However, this point is sometimes made when alluding to two particular features of owl vision; first that owls have high absolute sensitivity, and second, that owls may be unique among birds in having stereopsis. Certainly owls have high absolute visual sensitivity compared to other birds. For example, the absolute sensitivity of Tawny Owls *Strix aluco* is approximately 100 times greater than in Rock Doves *Columba livia* (Martin, 1977). However, high sensitivity is unlikely of itself to have driven the gaining of broad binocularity since as argued above binocular overlap in owls is no greater than in many bird species including passerines and some diurnal raptors, although it is broader than in doves. While there is evidence for stereopsis in owls (see section The Function of Binocular Vision in Birds) it is not clear how its presence should drive visual field configuration or indeed other general aspects of vision. Furthermore there is no evidence that stereopsis is used in owls' prey catching behavior. There are, in fact, good reason to believe that stereoscopic cues are not involved in the prey capture by owls. Resolution at low light levels in owls is low (Fite, 1973; Orlowski et al., 2012) and so any stereoscopic depth cues that might be available must be based upon this low resolution and are therefore unlikely to be available over a distance relevant to prey capture. Stereopsis is usually regarded as a rather slow process because it involves higher order processing, and the high sensitivity of owls may be achieved by relatively long temporal integration, as well as high spatial integration. These factor are likely to make stereopsis too slow to provide information on changing depth cues during a prey strike. It is also worth noting that even if owls do have access to relative depth cues based upon stereopsis that does not mean that they do not use flow-field information, alongside the direction and distance cues based upon hearing (Knudsen and Konishi, 1979). After all, humans have stereopsis, and sound location equal in accuracy to that of owls, but are highly dependent upon flow field information for the control of locomotion, especially time to contact a target.

Beyond high absolute sensitivity what is unusual about the vision of owls, compared with other birds, is the large blind area to the rear of the head. This possibly is the result of the elaborate outer ear structures in owls (Norberg, 1978) which are positioned just behind the eyes, and which are used for sound localization that is considerably more accurate than that of most other bird species (Klump, 2000). Because of these outer ear structures it would seem impossible for the visual fields of owls to be more extensive to the rear of the head. In essence the evolution of elaborate and large outer ears appears to have prevented owls evolving (or perhaps retaining from ancestral forms) more extensive visual coverage about their head.

Based upon this brief summary it would seem best not to regard owls, oilbirds, or other instances of nocturnally active bird species as posing a particular challenge to the general arguments presented in this review.

## The drivers of vision in birds

This review took as its start-point the phrase coined by Rochon-Duvigneaud (1943), “a bird is a wing guided by an eye,” and posed the question of whether flight is in fact the key task that has driven the evolution of avian vision. The above discussion has argued that there are in fact two key drivers of vision in birds and neither of them are concerned with the perceptual demands of flight. The primary driver is argued to be the control of bill position and the timing of its arrival at a target, the secondary driver is the task of detecting food items and predators. The control of bill position is based upon information derived from the optic flow-field in the binocular region that encompasses the bill. It is based upon information from the environment relatively close to the bird and depends upon relatively low spatial resolution. The detection of predators and food items is based upon information detected at a greater distance and depends upon regions in the retina with relatively high spatial resolution.

Interplay between these two key drivers of vision appear to be expressed in subtle interspecific variations in the vision of birds. The tasks of detecting predators and of placing the bill accurately, make contradictory demands upon vision and these have resulted in trade-offs in the form of visual fields and in the topography of retinal regions in which spatial resolution is enhanced (indicated by foveas and areas of high photoreceptor and ganglion cell densities).

The overall driver of frontal visual field characteristics appears to be the demand for the accurate positioning of the bill and the timing of its arrival at a target. This means that each eye must have a certain portion of its visual field which projects forwards and contralaterally (across the median sagittal plane of the head). The result of this is that in most birds there is a blind area behind the head which at any one moment constitutes an area in which predators cannot be detected. It is only in those few bird species which do not have to use vision to achieve precise control of bill position that natural selection has favored full visual coverage about the head.

Interspecific comparisons of visual fields between closely related species of ducks, shorebirds, and among emberizid passerines, have shown that small differences in foraging techniques can give rise to different perceptual challenges and these have resulted in subtle differences in visual fields even within the same genus. This suggests that vision can be subject to continuing and relatively rapid natural selection. This is perhaps not surprising given the inherent flexibility and individual differences in the structure of the optical system, retinal topography, and position in the skull, of vertebrate eyes (Figure 1).

It is important to note that patterns of photoreceptor and ganglion cell distribution in the retinas of birds (Lisney et al., 2012b; Coimbra et al., 2015) suggest considerable intraspecific variation in ganglion cell patterns, and that there is also evidence that these patterns can differ between the two eyes of the same individual (Mitkus et al., 2014) (Figure 2).

Together this variation in optics, retinal structure and eye position present a potent source of variation in vision that can be subject to natural selection at short time scales, much in the same way that individual differences in bill morphology can be the source of natural selection that underpin bill shape changes within a bird species over a short time scale (Grant and Grant, 2014).

Among birds there is a strong phylogenetic signal with respect to the maximum width of the binocular field, with passerine species showing broader widths than non-passerines, and within the passerines the broadest fields are found among the Corvidae (Troscianko et al., 2012). However, sample size with respect to the total number of passerines is small and more comprehensive species sampling of passerines, as well as non-passerines, may reveal some very interesting examples of the fine tuning of vision in birds.

The informational function of binocular vision in birds seems to lie not in binocularity *per se* (i.e., two eyes receiving slightly different information simultaneously about the same objects) but in the contralateral projection of the visual field of each eye (Martin, 2009). This ensures that each eye receives information from a symmetrically expanding optic flow-field centered close to the direction of the bill, and from this the crucial information of direction of travel and time-to-contact can be extracted, almost instantaneously.

In conclusion, it is proposed that the task of bill control is the key driver of bird vision, with predator detection also playing a key, but secondary role. The perceptual demands of flight are overshadowed by the demands of these two tasks. It seems that Rochon-Duvigneaud's (1943) assertion that “a bird is a wing guided by an eye” requires revision. From a sensory ecology perspective a bird is perhaps better characterized as “a bill guided by an eye” and that control of flight is achieved within constraints on visual capacity dictated primarily by the demands of foraging and, in particular, bill control.

## Author contributions

The author confirms being the sole contributor of this work and approved it for publication.

### Conflict of interest statement

The author declares that the research was conducted in the absence of any commercial or financial relationships that could be construed as a potential conflict of interest.
